# Predicting young Chinese consumers’ intentions to purchase Western brands: Structural model analysis

**DOI:** 10.1371/journal.pone.0267563

**Published:** 2022-05-06

**Authors:** Fei Long, Miraj Ahmed Bhuiyan, Norzalita Abd Aziz, Muhammad Khalilur Rahman

**Affiliations:** 1 UKM-Graduate School of Business, The National University of Malaysia, Bangi, Selangor, Malaysia; 2 School of Economics, Guangdong University of Finance and Economics, Guangzhou, China; 3 Faculty of Entrepreneurship and Business, Universiti Malaysia Kelantan, Kota Bharu, Kelantan, Malaysia; Universiti Malaysia Sabah, MALAYSIA

## Abstract

This study aims to investigate how young Chinese consumers make purchase intentions towards Western brands under the influence of two conflicting values and CSR, which is insufficiently discussed in the current literature. Both value-attitude-behavior (VAB) and consumer cultural theories are adopted to construct the research framework. Data was collected from undergraduate students studying at a public university located in Guangzhou via WeChat and Tencent QQ. A total of 314 usable responses were analyzed by the partial least squares structural equation modeling (PLS-SEM). The empirical findings indicated that cosmopolitanism has a significantly positive effect on brand attitudes and purchase intentions; ethnocentrism has a significantly negative effect on purchase intentions, but no significant impact on brand attitudes; and corporate social responsibility (CSR) initiatives positively affect brand attitudes rather than purchase intentions. The results also revealed that brand attitudes mediate the relationship between cosmopolitanism/CSR and purchase intentions, but it does not have a mediating effect on the relationship between ethnocentrism and purchase intentions. These findings provide essential insights to the body of knowledge of international marketing in emerging markets and shed light on understanding how young Chinese consumers make purchase decisions towards Western brands. The results are useful for Western brands to effectively adjust their marketing strategies and advertising/promoting campaigns for business development purposes in the Chinese market.

## Introduction

In the last two decades, China has gone through a major transformation from "World Factory" to "World Market" along with its economic rise. With an increasing disposable income, Chinese consumers are spending more on products and services. In 2019, China’s retail sales reached US$6.2 trillion, and the figure is only US$200 billion less than that of the USA, the largest consumer goods market in the world [1]. In addition, China’s economic ties with the world are generally getting closer and closer, so many young Chinese consumers are directly exposed to foreign cultures and products [2]. Under the influence of global consumer culture, young Chinese consumers have become westernized in relation to consumer beliefs, preferences, and behaviors [3]. Thus, international marketers postulate that young consumers are more acceptable to cosmopolitanism and less likely to refuse purchasing foreign brands out of ethnocentric tendencies in China [4], which is seen as a great market opportunity by Western brands.

Nevertheless, Western brands face challenges in this seemingly lucrative market as consumer values rapidly evolve amid China’s dramatic economic, social and cultural changes [3, 5]. Although empirical studies suggest that young Chinese consumers become more cosmopolitan and less ethnocentric [4], they rarely consider the rising economic/political/cultural tensions between China and the West [2]. The tensions are manifested by a series of international events and associated media coverage [6, 7]. One recent event is that some Western brands (e.g. Nike and Adidas) decided to stop buying Xinjiang cotton out of alleged human rights violations against minority Uyghurs. Consequently, they face a nationalism-driven backlash from Chinese consumers [8]. Tech-savvy young Chinese express their anger on social media and call for boycotts against these Western companies [9]. As stable socio-psychological factors, cosmopolitanism and ethnocentrism may influence consumers’ product selection process [10]. Prior relevant research mainly focuses on how the two factors’ influence attitudinal tendencies (e.g. brand loyalty) [11]. This study extends investigation towards their impacts on intentions to purchase Western brands in China. In addition, it is largely unknown how young Chinese consumers make purchasing decisions under the influence of two conflicting values.

Amid an increasing competition between China and the West (e.g. USA) in recent years, Western brands are facing more marketing challenges in China [2, 7]. Thus, marketing practitioners, policy-makers and researchers have made considerable attention on Western brands’ corporate social responsibility (CSR) initiatives in the Chinese market [12]. Some past studies indicate that CSR activities could mitigate consumers’ negative emotions towards foreign brands in China and eventually encourage Chinese consumers to patronize their products [13]. However, a few studies suggest that CSR initiatives may be ineffective [14], especially among consumers heavily influenced by nationalism as they tend to perceive foreign companies’ local CSR activities as insincere [15]. Moreover, it is inconclusive that CSR has a direct or indirect impact on purchase intentions [16, 17]. Therefore, whether and how CSR initiatives affect young Chinese consumers need to be empirically re-examined in the context of Western brands.

This study attempts to fill the knowledge gaps aforementioned in marketing literature in the context of Western brands in China. The research objectives of this study are to investigate how consumer values (i.e. ethnocentrism and cosmopolitanism) and CSR initiatives influence young Chinese consumers’ (university students) intentions to purchase Western brands through attitudes. Therefore, this research makes substantial contributions to international marketing twofold. First, this study enhances our understanding of how ethnocentric and cosmopolitan values influence young Chinese consumers’ intentions to purchase Western brands via attitudes. Second, our research verifies CSR initiatives’ role in mitigating consumers’ negative attitudes (emotions) against Western brands.

## Literature review

### Theoretical underpinning to purchase intentions

Value-attitude-behavior (VAB) theory [18] is one of the most widely used theory in explaining consumers’ decision-making process and predicting their consequent consumption behaviors [15, 19, 20]. In this study, we have used the concept of VAB theory [18] to evaluate the predicting of young Chinese consumers’ brand attitude towards purchase intention of Western brands. Both values and attitudes are considered significant factors influencing behavioral intentions and actual behaviors [21]. In this study, value is defined as a young consumer’s fundamental standard utilized for evaluating the external environment and internalized standard directs their attitudinal and behavioral predispositions [22]. Engel et al. [23] argue that each consumer has a value system that works as a desirable criterion to guide his/her behaviors. With regard to attitude, Ajzen [24] defined it as “the degree to which a person has a favorable or unfavorable evaluation or appraisal of the behavior in question.” In short, it refers to a relatively stable feeling responding to an object or behavior based on behavioral beliefs [25].

Regarding the relationship among values, attitudes and behaviors, Milfont et al. [26] state that VAB is a sequential hierarchy model, and the influence of an individual’s abstract values is imposed on a certain behavior via his/her attitudes toward the behavior. Initially, Homer and Kahle [18] proposed a hierarchical cognitive model connecting more abstract cognitions (values), mid-range cognitions (attitudes) and particular intentions/behaviors. In their own words, "the influence should theoretically flow from abstract values to mid-range attitudes to specific behaviors." [18]. The influence of values and attitudes on intentions/behaviors and the validity of the VAB model have been empirically supported by many prior studies in different contexts [27–29]. VAB provides a theoretical ground to examine the interplay of values, attitudes and behavioural intentions to purchase Western brands. Therefore, the overall response towards Western brands is mainly determined by one’s values, and the relationship is mediated by the individual’s attitudes [19].

In addition, consumer cultural theory (CCT) proposed by Arnould and Thompson [30] is adopted for enhancing the robustness of this research’s framework [31]. Consumer culture is initiated by commercially produced images, symbols, objects and their attached information to consumers. Then, it is systematically constructed by the interactions and even conflicts of market-oriented information, consumer identities, marketplace social situations and social structures [32]. Besides, consumer culture is an interconnected network that is composed of local cultures and transnational capital-driven global cultures [33]. Furthermore, consumer culture frames consumers’ feelings, thoughts, and emotions to form certain behavioral tendencies and patterns that are divergent from other people [23, 32]. Although CCT, as a collection of theoretical perspectives rather than an ultimate theory, does not directly establish nomothetic models, it facilitates researchers and practitioners comprehending the complex and dynamic relationship among cultural values, consumer behaviors and the marketplace [30, 34]. Based on prior research, ethnocentrism and cosmopolitanism are identified as critical consumer values determining consumers’ decision-making process and consumption behaviors [4, 5, 7, 35]. In light of the aforementioned, ethnocentrism, cosmopolitanism and attitudes are considered as determinants of intentions to purchase Western brands among young Chinese consumers in this research.

### Ethnocentrism

Ethnocentrism refers to a personal belief in relation to the question whether it is appropriate to purchase foreign products from an economic perspective [36]. Ethnocentric consumers usually prefer domestic over foreign products as they "view of things in which one’s own group is the center of everything, and all others are scaled and rated with reference to it" [37], which makes ethnocentrism a cognitive tendency favoring in-group membership and detesting out-group members based on biased evaluation [4, 38, 39]. In addition, Shimp and Sharma [40] argue that ethnocentric consumers generally associate purchasing foreign products to unpatriotic behavior because it hurts their own economy. Due to the potential adverse impacts on the local economy, they emotionally evaluate foreign products (brands). Therefore, they are willing to sacrifice the maximization of self-interest to protect their country’s interests [41].

It has been constantly confirmed that ethnocentrism is a significant factor in consumers’ purchase decisions regarding local or foreign products [7, 35, 41–43]. For example, Qing et al. [44] found that ethnocentrism positively influences Chinese consumers’ attitudes toward domestic fruits [39]. In addition, Thomas et al. [45] revealed that ethnocentrism negatively affects Indian consumers’ attitudes towards foreign brands. Furthermore, Blazquez-Resino et al. [42] affirmed that ethnocentric consumers tend to have positive behaviors regarding attributes assessment and purchase intention towards local products. Meanwhile, it has opposite effects on foreign products [46].

However, there are contradictory findings from a few prior studies. Chakraborty and Sadachar [47] found that ethnocentrism does not have a significant and negative effect on attitudes towards Western brands though it negatively influences purchase intentions. Meanwhile, Liu & Hong [11] concluded that ethnocentrism has no significant impact on purchase intentions with regard to cross-border E-commerce in China, which is supported by Nguyen and Pham [38]. Han and Guo [48] also argue that ethnocentrism is a poor predictor of intentions to purchase foreign brands based on data from 220 Chinese millennials. Given the inconsistency, the interplay between ethnocentrism, attitudes and purchase intentions towards Western brands should be re-examined, especially in the context of young Chinese consumers. Therefore, we postulated that:

H1: Ethnocentrism negatively influences brand attitudes towards Western brands among young Chinese consumers.
H2: Ethnocentrism negatively influences purchase intention towards Western brands among young Chinese consumers.
H3: The negative relationship between ethnocentrism and purchase intention is mediated by attitudes towards Western brands among young Chinese consumers.

### Cosmopolitanism

Cosmopolitanism is defined as cognitive tendencies to direct individuals to explore areas beyond the boundary of their own culture, community, and society [4]. Cosmopolitan individuals usually are not biased towards other groups but evaluate their own and other groups on merits [10]. Riefler et al. [49] argue that cosmopolitan consumers are open-minded towards foreign cultures, and therefore, they are likely to seek and receive comprehensive information about foreign brands. As "sophisticated" consumers, they tend to objectively judge foreign brands based on their quality attributes [40]. In addition, cosmopolitan consumers are inclined to see themselves as global citizens, so they are likely to have more favorable attitudinal and behavioral responses towards foreign brands by comparison to non-cosmopolitan ones [10].

Furthermore, consumers of developing countries may have a higher level of cosmopolitan tendency with regard to consumption behaviors as they perceive that products of Western brands have a better quality than that of domestic ones [38, 50]. Besides, young Chinese consumers are becoming more individualistic and thus are less affected by external social influences [31]. As a result, they are arguably more cosmopolitan than older generations [3, 50]

The positive association between cosmopolitanism and consumers’ attitudes and purchase intentions towards Western brands is supported by previous studies [31, 38, 43, 51]. For example, Chakraborty and Sadachar [47] found that cosmopolitanism positively influences Indian consumers’ intentions to purchase Western apparel brands; Nguyen and Pham [38] revealed that cosmopolitanism has a positive effect on purchase intentions towards foreign products among young Vietnamese consumers; Liu & Hong [11] confirmed that Chinese consumers with high cosmopolitanism have more favorable attitudes towards foreign E-commerce channels, and thus tend to purchase products from these channels. However, Zeugner-Roth et al. [10] concluded that cosmopolitanism has no significant impact on consumers’ preference towards foreign products, which suggests an inconclusive interplay among consumer cosmopolitanism, brand attitudes, and purchase intentions. Thus, we proposed that:

H4: Cosmopolitanism positively influences brand attitudes towards Western brands among young Chinese consumers.
H5: Cosmopolitanism positively influences purchase intentions towards Western brands among young Chinese consumers.
H6: The positive relationship between cosmopolitanism and purchase intention is mediated by attitudes towards Western brands among young Chinese consumers.

### Corporate social responsibility (CSR)

The VAB model is constantly confirmed effective in predicting consumption behaviors, but it only focuses on consumers’ internal values and underestimates some external factors, such as corporate social responsibility (CSR) [15, 19]. Therefore, CSR is combined with the VAB model to better understand young Chinese consumers’ attitudinal and behavioral tendencies pertaining to Western brands.

CSR is described as a firm’s commitment to conduct business ethically and make economic, social and environmental contributions in a sustainable way to various stakeholders, such as consumers, suppliers, communities and governments [15, 16, 52]. In the past few decades, researchers and practitioners have paid considerable attention to a firm’s CSR because it is believed that CSR facilitates a firm to achieve favorable organization and employee related outcomes, including organizational performance [53], corporate financial performance [54] and employee engagement [55]. Comparatively, there is insufficient research examining the effects of CSR from a consumer perspective, such as consumer purchase intentions [52].

Nevertheless, it is found that a firm’s CSR and related activities could reduce customers’ negative emotions and also increase customers’ positive evaluations toward the firm and its products [13]. As a result, consumers are more likely to purchase products from a firm with positive CSR perceptions [15]. Meanwhile, Olšanová et al. [16] revealed that CSR initiatives directly impact purchase intentions [56]. However, Ramesh et al. [17] concluded that CSR activities have no direct impacts on purchase intentions but indirect effects through brand attitudes. Thus, the relationship between CSR initiatives, brand attitudes, and purchase intentions need to be reexamined. In foreign products or Western brands, CSR may help them be perceived as domestic if these firms are committed to contributing to local society [13]. Then, consumers are more likely to purchase from them [57]. Therefore, we postulated that:

H7: CSR initiatives positively influence brand attitudes towards Western brands among young Chinese consumers.
H8: CSR initiatives positively influence purchase intentions towards Western brands among young Chinese consumers.
H9: The positive relationship between CSR initiatives and purchase intentions is mediated by attitudes towards Western brands among young Chinese consumers.

### Brand attitudes

The VAB model postulates that an individual’s attitudes positively influences his/her behavioral intentions, and the proposed relationship has been often supported with empirical evidence [20–22, 26, 27]. Han et al. [22] examined consumers’ decision-making process for eco-cruise products. They found that attitudes positively influenced different behavioral intentions, including word-of-mouth, purchase, and sacrifice intentions. Similarly, Tajeddini et al. [19] explored consumers’ purchase intentions of Airbnb and hotel accommodations, and they verified the positive association between attitudes and behavioral intentions. Moreover, Jung et al. [58] tested Chinese consumers’ preferences to purchase sustainable apparel products, and it is found that attitudes have a strong positive effect on purchase intentions [31]. Therefore, we postulated that:

H10: Brand attitudes positively influence purchase intentions towards Western brands among young Chinese consumers.

**Fig 1:**
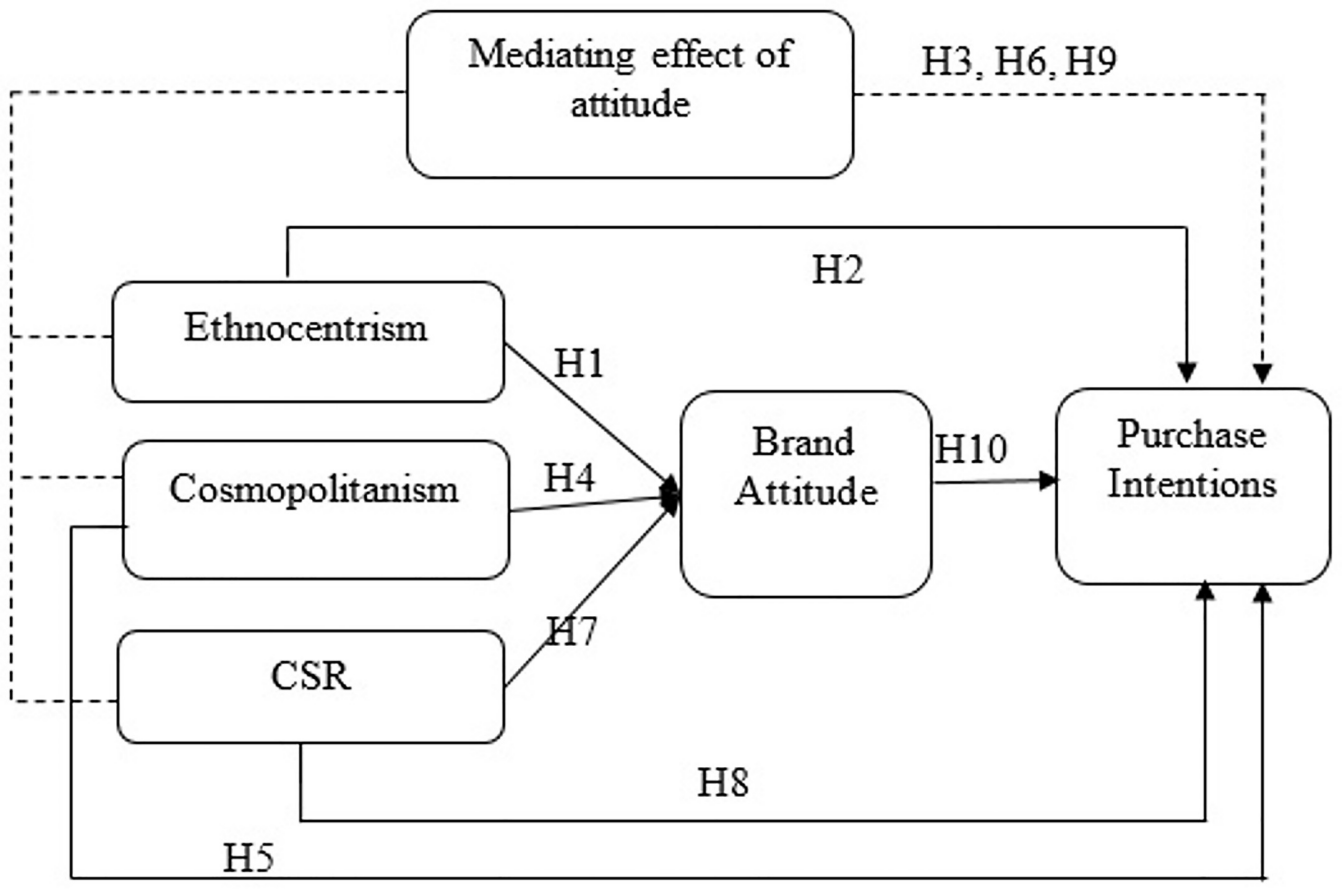
Conceptual framework: Author’s elaboration.

Based on the review of the literature and theoretical background, we have proposed a conceptual framework (Fig 1).

## Methodology

An online self-administered questionnaire was developed to examine the proposed research framework concerning young Chinese consumers’ intentions to purchase Western brands. All the 23 measurement items were adopted/adapted from previous studies related to consumer marketing [59]. All items were assessed by a 7-point Likert scale from 1 (strongly disagree) to 7 (strongly agree) except the items of the criterion variable (i.e. purchase intentions) that were measured by a 5-point Likert scale from 1 (strongly disagree) to 5(strongly agree). The rationale to use different scales to predictive and criterion variables is to minimize common method bias with data collected from a single source [60]. The latent variables and their associated items of the research are shown in Table 1.

**Table 1:** Questionnaire design.

Constructs	Item No.	Measurement items	Source
**Ethnocentrism (EC)**	EC1	Chinese should not buy Western brands, because this hurts Chinese business and causes unemployment.	[39]
	EC2	It may cost me in the long-run but I prefer to support domestic brands.	
	EC3	It should not be allowed to put Western brands in our markets.	
	EC4	Western brands should be taxed heavily to reduce their entry into China.	
	EC5	We should buy from Western brands only when we cannot obtain some products from domestic brands.	
**Cosmopolitanism (CO)**	CO1	I am interested in learning more about people who live in other countries.	[4]
	CO2	I enjoy exchanging ideas with people from other cultures or countries.	
	CO3	I like to learn about foreign cultures.	
	CO4	I feel comfortable thinking about international marriage.	
	CO5	It is desirable for foreigners to take part in economic activities in China.	
**CSR Initiatives (CSR)**	CSR1	Western brands are committed to helping to solve social problems.	[15]
	CSR2	Local communities benefit from Western brands’ CSR activities.	
	CSR3	Western brands are interested in serving public interest in China.	
	CSR4	Western brands give back to the communities.	
	CSR5	Western brands are socially responsible.	
**Brand Attitudes (BA)**	BA1	Western brands are good.	[61]
	BA2	Western brands are favorable.	
	BA3	Western brands are positive.	
	BA4	Western brands are of high quality.	
**Purchase Intentions (PI)**	PI1	I am very likely to buy Western brands.	[48]
	PI2	I would like to own Western brands.	
	PI3	I am interested in buying Western brands.	
	PI4	I would recommend Western brands to others.	

The questionnaire was initially developed in English, and it was translated into Chinese by the leading author who used to work as a professional Chinese-English translator. Then, the Chinese version was back-translated by a native Chinese speaker who taught English at university [62]. By comparing the original questionnaire and the back-translated English version, we found no significant differences. Before distributing the questionnaire, we pre-tested it with five young Chinese consumers (students from a public university of China). The five respondents did not report any problems in understanding the Chinese version of the questionnaire.

The online questionnaire was created at Tencent Wenjuan (URL: https://wj.qq.com/), one of China’s most popular survey platforms. From 22 May to 22 June 2021, the online survey’s URL link was sent to 1,000 undergraduate students studying at a public university located in Guangzhou via WeChat and Tencent QQ. As one of the largest Chinese metropolises, Guangzhou is well connected with the world through trade. Thus, Guangzhou residents (e.g. students) are exposed to Western cultures and products [63]. By the end of June 2021, 314 usable responses had been collected with a response rate of 31.4%. G*Power 3.1 statistical tool was used for evaluating sample size of this study. Based on the conceptual model of this study, we have considered four predictors (e.g. ethnocentrism, cosmopolitanism, CSR, brand attitudes, and consumers’ purchase intentions towards Western brands). The G*Power test results indicate that the minimum sample size is 129, which provides actual statistical power of 0.95. In this study, we have a sample size that is greater than 129. Besides, Using the partial least square (PLS) method, the minimum sample size is required 100 [64]. In this study, we have collected 314 valid responses from young Chinese consumers, exceeding the minimum sample size requirement. Thus, the sample size of this study is acceptable and adequate for the analysis.

## Empirical results

### Descriptive statistics for scale items

The mean value, standard deviation, kurtosis and skewness of each scale item are shown in Table 2. With regard to kurtosis and skewness, the values are within the range of ±7 and ±2 respectively. Thus, the data of this research is normally distributed [65]. Although PLS-SEM used by this research is able to handle non-normally distributed data, recent literature suggests that extremely non-normal data is problematic in assessing parameters’ significance [66]. Meanwhile, the respondents generally have a stronger cosmopolitanism than ethnocentrism, and they have slightly negative CSR perceptions and attitudes towards Western brands based on the mean values of the latent variables. It is slightly higher than the neutral point (3.210 out of 5) about their purchase intentions.

**Table 2:** Descriptive analysis.

Construct/Associated Items	Mean value	Standard deviation	Excess Kurtosis	Skewness
**Ethnocentrism (EC)**	**3.018**			
EC1	2.930	1.401	-0.307	0.160
EC2	3.904	1.475	0.286	-0.032
EC3	2.089	1.271	-0.702	0.729
EC4	3.051	1.332	0.029	0.223
EC5	3.118	1.461	-0.654	0.016
**Cosmopolitanism (CO)**	**4.839**			
CO1	5.191	1.329	-0.195	-0.248
CO2	5.226	1.320	0.351	-0.448
CO3	5.038	1.274	0.178	-0.165
CO4	4.194	1.679	-0.448	-0.200
CO5	4.545	1.274	0.418	0.113
**CSR Initiatives (CSR)**	**3.714**			
CSR1	3.350	1.194	0.615	-0.240
CSR2	3.943	0.989	2.245	-0.063
CSR3	3.844	1.043	2.052	-0.124
CSR4	3.799	1.010	1.771	-0.353
CSR5	3.637	1.121	1.180	-0.407
**Brand Attitudes (BA)**	**3.624**			
BA1	3.576	1.039	1.388	-0.796
BA2	3.634	1.023	1.597	-0.580
BA3	3.561	0.986	1.641	-0.850
BA4	3.723	1.060	1.307	-0.572
**Purchase Intentions (PI)**	**3.210**			
PI1	3.315	0.748	1.400	0.328
PI2	3.162	0.724	1.767	0.349
PI3	3.210	0.719	1.448	0.283
PI4	3.153	0.746	1.862	0.021

### Confirmatory Factor Analysis (CFA)

To assess the proposed hypotheses of this research, CFA was conducted using SmartPLS (version 3.3.3). The authors followed the procedure suggested by Hair et al. [66] for data analysis and reporting of measurement and structural model. With regard to the measurement model, both reliability and validity are confirmed. As shown in Table 3, all scale items’ factor loadings are higher than 0.708 except EC1, EC2 and CO4. In addition, the AVE and CR values of ethnocentrism (EC) and cosmopolitanism (CO) are greater than their benchmarks (i.e. 0.50 and 0.70), respectively. Therefore, the three scale items are kept. Concerning indicator consistency reliability, the authors referred to rho_A rather than Cronbach’s alpha and CR. Dijkstra & Henseler [67] explained that Cronbach’s alpha usually underestimates true reliability, and CR usually overestimates true reliability. The rho_A value of the latent variables ranges between 0.70 and 0.95, so this research’s internal consistency is confirmed [68]. With regard to convergent validity and discriminant validity, AVE and HTMT (see Table 4) are used as reference, and the latent variables’ AVE and HTMT are higher than the thresholds [66]. The measurement model is established based on the indicator loadings, rho_A, AVE, and HTMT values.

**Table 3:** Assessment results of the measurement model.

Construct/Associated Items	Loading	Cronbach’s α	rho_A	CR	AVE
**Ethnocentrism (EC)**		0.780	0.806	0.849	0.531
EC1	0.677				
EC2	0.615				
EC3	0.797				
EC4	0.780				
EC5	0.757				
**Cosmopolitanism (CO)**		0.819	0.846	0.878	0.598
CO1	0.871				
CO2	0.868				
CO3	0.858				
CO4	0.489				
CO5	0.711				
**CSR Initiatives (CSR)**		0.852	0.871	0.892	0.623
CSR1	0.768				
CSR2	0.751				
CSR3	0.765				
CSR4	0.844				
CSR5	0.815				
**Brand Attitudes (BA)**		0.897	0.898	0.928	0.764
BA1	0.854				
BA2	0.883				
BA3	0.902				
BA4	0.857				
**Purchase Intentions (PI)**		0.937	0.939	0.955	0.84
PI1	0.888				
PI2	0.926				
PI3	0.937				
PI4	0.915				

Note: CR = Composite Reliability, AVE = Average Variance Explained.

**Table 4:** Discriminant validity.

	BA	CSR	CO	EC
CSR	0.590			
CO	0.351	0.346		
EC	0.109	0.221	0.300	
PI	0.487	0.311	0.390	0.214

Note: Discriminant validity established at HTMT _0.90_.

Before examining the structural model, we checked the variance inflation factor (VIF) of this research. Although the vertical collinearity is ruled out as the discriminant validity is confirmed, lateral collinearity (predictor-criterion collinearity) may distort the significance of the causal effects of the research [69]. As shown in Table 5, all the VIF values are less than the benchmark of 3.3, indicating that there is no multicollinearity issue in this study [66].

**Table 5:** Variance inflation factor.

	BA	PI
BA		1.460
CSR	1.185	1.524
CO	1.218	1.260
EC	1.153	1.156

The structural model was analyzed by utilizing a bootstrapping procedure with 5000 resamples [66]. This research model has a substantial level of predictive accuracy on the two endogenous variables as the R^2^ values of brand attitudes and purchase intentions are 0.315 and 0.286 respectively [70]. Besides, the research model has a predictive relevance as the Q^2^ values of brand attitudes and purchase intentions are 0.223 and 0.221, respectively [66]. With regard to the path analysis, the authors assessed path coefficient, t-value, p-value, effect size (f^2^) and confidence intervals [66]. Based on Table 6, H1 and H8 are rejected, and H2, H4, H5, H7, and H10 are supported. In relation to the assessment of mediation analysis, this research applied “bootstrapping the indirect effect” rather than Baron and Kenny’s method because bootstrapping is one of the most powerful methods for testing mediating effect [66]. Preacher and Hayes [71] explained that direct effect is not necessary for mediation analysis and what matters is indirect effect. The results summary of the bootstrapping analysis is shown in Table 7. It is found that H3 is rejected; H6 and H9 are supported.

**Table 6:** Path coefficient.

Hypothesis	Relationship	Path Coefficient	T Value	P Value	Effect Size	LL	UL	Decision
H1	EC -> BA	0.044	0.663	0.254	0.002	-0.064	0.15	Rejected
H2	EC -> PI	-0.202	3.492	0.000	0.049	-0.303	-0.117	Supported
H4	CO -> BA	0.169	2.817	0.002	0.034	0.071	0.27	Supported
H5	CO -> PI	0.159	2.317	0.010	0.028	0.048	0.273	Supported
H7	CSR -> BA	0.482	8.991	0.000	0.287	0.395	0.571	Supported
H8	CSR -> PI	0.074	1.000	0.159	0.005	-0.047	0.197	Rejected
H10	BA -> PI	0.380	6.201	0.000	0.139	0.279	0.483	Supported

Notes: LL (lower limit) and UL (upper limit) at 95 percent confidence interval.

**Table 7:** Mediation effects.

Hypothesis	Relationship	Path Coefficient	T Value	P Value	LL	UL	Decision
H3	EC -> BA -> PI	0.017	0.655	0.256	-0.024	0.058	Rejected
H6	CO -> BA -> PI	0.064	2.419	0.008	0.025	0.111	Supported
H9	CSR -> BA -> PI	0.183	4.814	0.000	0.126	0.248	Supported

Notes: LL (lower limit) and UL (upper limit) at 95 percent confidence interval.

Based on the path analysis results in Tables 6 and 7, it is found that cosmopolitanism has a positive effect on brand attitudes (Beta = 0.169, t = 2.817, p = 0.002, f^2^ = 0.034, LL = 0.071, UL = 0.270) and purchase intentions (Beta = 0.159, t = 2.317, p = 0.010, f^2^ = 0.028, LL = 0.048, UL = 0.273) towards Western brands among young Chinese consumers, and brand attitudes mediate the positive relationship between cosmopolitanism and purchase intentions (Beta = 0.064, t = 2.419, p = 0.008, LL = 0.025, UL = 0.111). Meanwhile, ethnocentrism has a negative effect on purchase intentions (Beta = -0.202, t = 3.492, p = 0.000, f^2^ = 0.049, LL = -0.303, UL = -0.117), but it does not have a negative effect on brand attitudes and brand attitudes does not mediate the negative relationship between ethnocentrism and purchase intentions as we expected. With regard to CSR, it has a positive effect on brand attitudes (Beta = 0.482, t = 8.991, p = 0.000, f^2^ = 0.287, LL = 0.395, UL = 0.571), but it doesn’t influence purchase intentions directly. In addition, brand attitudes mediate the positive relationship between CSR and purchase intentions (Beta = 0.183, t = 4.814, p = 0.000, LL = 0.126, UL = 0.248).

## Discussion and implications

In this study, we intended to understand how consumer values influence consumption behaviors in China by empirically examining the effects of ethnocentrism and cosmopolitanism on young Chinese consumers’ attitudes and purchase intentions towards Western brands. In addition, we also sought to address the question of whether a firm’s CSR initiatives could change consumers’ attitudes and purchase intentions.

According to the mean values of the latent variables, young Chinese consumers’ cosmopolitanism (4.839 out of 7) is much more salient than ethnocentrism (3.018 out of 7), which indicates that young Chinese consumers are willing to expose themselves to foreign cultures, products and brands [50]. This may be because young Chinese become more individualistic in making purchase decisions [4], and they care more about their own interests than group ones [3, 50]. Meanwhile, their purchase intentions are moderate (3.210 out of 5). However, young Chinese consumers do not have very positive evaluations of Western brands as their brand attitudes (3.624 of 7) and CSR perceptions (3.714 out of 7) are slightly weak. This may suggest that Western brands have not done enough CSR initiatives in China.

Significantly, this research generally reaffirms that consumers’ abstract values influence their mid-range attitudes and specific behavioral intentions, but ethnocentrism, as an abstract value, does not affect young Chinese consumers’ attitudes towards Western brands. The inconsistent relationships between consumer values and consumption preferences suggest the complexity of consumer culture constantly being shaped and reshaped by local and capital-driven global cultures [33]. Young Chinese consumers tend to have a mature and sophisticated product evaluation system, which facilitates them forming a relatively objective assessment towards product quality and attributes [48]. Besides, it is found that a firm’s CSR does not directly influence purchase intentions as previous studies indicated [56], but it has an indirect impact on purchase intentions via brand attitudes [17]. These findings shed light on the consumption behaviors of young Chinese towards Western brands by providing some relevant academic and managerial implications.

### Theoretical contributions

This research constructs an extended VAB model incorporating consumer culture theory (i.e. ethnocentrism and cosmopolitanism) and CSR to advance our understanding of consumption behaviors and expand the current literature of international marketing in the context of China. This extended VAB model has substantial power in explaining young Chinese consumers’ attitudinal and behavioral tendencies to purchase Western brands. With an increasing disposable income, Chinese consumers and their consumption behaviors have attracted much attention from researchers and practitioners [2, 5]. However, previous studies mainly focus on consumers’ socio-psychological factors, and little research has been conducted to comprehend consumers’ attitudinal and behavioral propensities.

Firstly, cosmopolitanism has a significantly positive effect on brand attitudes and purchase intentions, which is consistent with most previous studies concerning consumer cosmopolitanism [11, 31, 38, 47]. These findings mean that cosmopolitan consumers are more likely to form favorable affective evaluations (i.e. brand attitudes) and purchase intentions towards Western brands. More importantly, brand attitudes have a direct impact on purchase intentions, and they mediate the positive relationship between cosmopolitanism and purchase intentions [21, 24, 51], which is also supported by previous studies [11, 31, 38, 47]. Thus, the importance of cosmopolitanism in predicting attitudes and purchase intentions towards Western brands has been confirmed among young Chinese consumers.

Secondly, ethnocentrism has no significant influence on consumers’ brand attitudes. In addition, brand attitudes do not mediate the negative relationship between ethnocentrism and purchase intentions. These findings imply that young Chinese consumers of high ethnocentrism are less likely to purchase Western brands, but their ethnocentric propensity will not negatively affect their attitudes towards Western brands. The insignificant relationship between ethnocentrism and brand attitudes might be attributed to the fact that Chinese consumers have much knowledge/information about Western brands [46], so they tend to have logic and subjective assessments on product attributes. They probably will not form negative attitudes towards Western brands simply because of their ethnocentric tendencies [39]. Although ethnocentrism is moderately low among young Chinese consumers, ethnocentrism does have a significantly negative effect on purchase intentions, which may be caused by the social obligations (subjective norms) highlighting national interests by avoiding Western products, especially when China and Western countries are having various conflicts [6, 7, 9].

Thirdly, CSR initiatives positively affect brand attitudes, but they do not significantly influence purchase intentions as we expected. Nevertheless, CSR initiatives indirectly influence purchase intentions via brand attitudes [17]. The findings suggest that young Chinese consumers are more likely to form favorable attitudes towards Western brands when they perceive more CSR initiatives from these brands. Alternatively, CSR initiatives effectively alleviate consumers’ negative emotions towards foreign businesses [13]. Western brands’ CSR initiatives do not directly increase purchase intentions, but they do facilitate young Chinese consumers forming positive evaluations of Western brands. Then, they tend to purchase Western brands due to favorable brand attitudes [22, 57].

### Practical implications

This research is beneficial to international marketing managers with regard to emerging markets (e.g. China) in a few important ways. This study suggests that young Chinese consumers’ cosmopolitanism outshines their ethnocentrism, and they are generally open-minded to foreign products or brands. However, the influence of ethnocentrism seems stronger than that of cosmopolitanism in relation to purchasing intentions. In addition, young Chinese consumers are very likely to boycott Western brands due to strong patriotic obligations when China and Western countries are having conflicts even though they do not have negative evaluations towards Western brands. Therefore, Western brands are advised to keep monitoring ethnocentrism and related international events in China. Meanwhile, international marketers should identify market segments based on consumers’ ethnocentrism and cosmopolitanism levels, and target them with different marketing strategies. For example, marketing mangers should emphasize product attributes and blur country of origin when local consumers have negative sentiments towards countries associated with the products/brands. Moreover, Western brands are advised to carry out more CSR initiatives and brand themselves as socially, economically and environmentally responsible in China. Although CSR initiatives do not directly influence purchase intentions, they could improve consumers’ attitudes and indirectly influence purchase intention.

### Limitations and suggestions for future research

This study provides important theoretical and practical implications, but it has a few limitations as well. First, the research examined purchase intentions rather than actual purchase behaviors. Future research should investigate the actual behaviors as Ajzen [25] explains that people may not conduct a certain behavior precisely in line with their related behavioral intention. Second, the findings of the research may not be generalized because the data was collected from undergraduates of Guangzhou. Individuals’ consumer values, consumption preferences, and behaviors may vary conspicuously in different regions of China due to an imbalance of economic development [72]. Thus, future studies should collect data from the different areas and groups to represent young Chinese consumers. Third, other essential factors, such as individualism and animosity [31, 48] could significantly influence brand attitudes and purchase intentions. Future research may include these factors to enhance the predictive power of the extended VAB model of the present study. Fourth, this only examined young Chinese consumers, and generational differences may exist. Thus, future studies are advised to compare consumers from different generational cohorts to see whether there are disparities or similarities with regard to intentions to purchase Western brands.

## Supporting information

S1 Data (CSV)

## References

[1] Tang, F. (2020). China to overtake US to become world’s top consumer goods market ’very soon’. South China Morning Post. https://www.scmp.com/economy/china-economy/article/3111954/china-overtake-us-become-worlds-top-consumer-goods-market

[2] Lee Y. H., Wei C. F., Lee B. C., Cheng Y. Y., & Chen Y. (2021). Consumer brand engagement in the US–China trade war. Asia Pacific Journal of Marketing and Logistics. doi: 10.1108/APJML-03-2020-0162

[3] Neuliep J. W. (2020). Intercultural communication: A contextual approach (6^th^ ed.). Sage Publications.

[4] Han C. M. (2017). Cosmopolitanism and ethnocentrism among young consumers in emerging Asia. Asia Pacific Journal of Marketing and Logistics, 29(2), 330–346. doi: 10.1108/APJML-07-2016-0113

[5] Rašković M., Ding Z., Hirose M., Žabkar V., & Fam K. S. (2020). Segmenting young-adult consumers in East Asia and Central and Eastern Europe–The role of consumer ethnocentrism and decision-making styles. Journal of Business Research, 108, 496–507.

[6] Heinberg M. (2017). Outbreaks of animosity against the West in China: effects on local brand consumption. International Marketing Review, 34(4), 514–535. doi: 10.1108/IMR-07-2014-0222

[7] Lee H. M., Chen T., Chen Y. S., Lo W. Y., & Hsu Y. H. (2020). The effects of consumer ethnocentrism and consumer animosity on perceived betrayal and negative word-of-mouth. Asia Pacific Journal of Marketing and Logistics, 33(3), 712–730. doi: 10.1108/APJML-08-2019-0518

[8] Friedman, V., & Paton, E. (2021). What Is Going On With China, Cotton and All of These Clothing Brands? The New York Times. https://www.nytimes.com/2021/03/29/style/china-cotton-uyghur-hm-nike.html

[9] DW (2021). Xinjiang cotton boycott leaves Western brands reeling. https://www.dw.com/en/xinjiang-cotton-boycott-leaves-western-brands-reeling/a-57130450.

[10] Zeugner-Roth K. P., Žabkar V., & Diamantopoulos A. (2015). Consumer ethnocentrism, national identity, and consumer cosmopolitanism as drivers of consumer behavior: A social identity theory perspective. Journal of international marketing, 23(2), 25–54.

[11] Liu C., & Hong J. (2020). The Effects of Consumer Cosmopolitanism and Consumer Ethnocentrism on Cross-border E-commerce in China. Journal of International Trade & Commerce, 16(3), 43–57.

[12] Chu S. C., Chen H. T., & Gan C. (2020). Consumers’ engagement with corporate social responsibility (CSR) communication in social media: Evidence from China and the United States. Journal of Business Research, 110, 260–271.

[13] Kim J. H. (2019). Animosity and switching intention: moderating factors in the decision making of Chinese ethnic diners. Cornell Hospitality Quarterly, 60(2), 174–188.

[14] Achabou M. A. (2020). The effect of perceived CSR effort on consumer brand preference in the clothing and footwear sector. European Business Review, 32(2), 317–347. doi: 10.1108/EBR-11-2018-0198

[15] Han C. M., Kim K. A., & Nam H. (2019a). Can corporate philanthropy change consumers’ perceptions of Japanese multinationals and reduce animosity toward them?. Asia Pacific Journal of Marketing and Logistics, 32(1), 65–85. doi: 10.1108/APJML-09-2018-0383

[16] Olšanová K., Ríos A. E., Cook G., Král P., & Zlatić M. (2021). Impact of the awareness of brand-related CSR activities on purchase intention for luxury brands. Social Responsibility Journal, ahead-of-print(ahead-of-print). doi: 10.1108/SRJ-10-2020-0398

[17] Ramesh K., Saha R., Goswami S., & Dahiya R. (2019). Consumer’s response to CSR activities: Mediating role of brand image and brand attitude. Corporate Social Responsibility and Environmental Management, 26(2), 377–387.

[18] Homer P. M., & Kahle L. R. (1988). A structural equation test of the value-attitude-behavior hierarchy. Journal of Personality and social Psychology, 54(4), 638–646.

[19] Tajeddini K., Rasoolimanesh S. M., Gamage T. C., & Martin E. (2021). Exploring the visitors’ decision-making process for Airbnb and hotel accommodations using value-attitude-behavior and theory of planned behavior. International Journal of Hospitality Management, 96, 102950. doi: 10.1016/j.ijhm.2021.102950

[20] Cheung M. F., & To W. M. (2019). An extended model of value-attitude-behavior to explain Chinese consumers’ green purchase behavior. Journal of Retailing and Consumer Services, 50, 145–153.

[21] Hurst M., Dittmar H., Bond R., & Kasser T. (2013). The relationship between materialistic values and environmental attitudes and behaviors: A meta-analysis. Journal of Environmental Psychology, 36, 257–269.

[22] Han H., Hwang J., Lee M. J., & Kim J. (2019b). Word-of-mouth, buying, and sacrifice intentions for eco-cruises: Exploring the function of norm activation and value-attitude-behavior. Tourism Management, 70, 430–443.

[23] Engel J.F., Blackwell R.D., & Miniard P.W. (1995). Consumer Behavior (8th ed.). The Dryden Press, Fort Worth.

[24] Ajzen I. (1991). The theory of planned behavior. Organizational Behavior and Human Decision Processes, 50, 179–211.

[25] Ajzen I. (2020). The theory of planned behavior: Frequently asked questions. Human Behavior and Emerging Technologies, 2(4), 314–324.

[26] Milfont T. L., Duckitt J., & Wagner C. (2010). A cross-cultural test of the value–attitude–behavior hierarchy. Journal of Applied Social Psychology, 40(11), 2791–2813.

[27] Kim M. J., Hall C. M., & Kim D. K. (2020). Predicting environmentally friendly eating out behavior by value-attitude-behavior theory: does being vegetarian reduce food waste?. Journal of Sustainable Tourism, 28(6), 797–815.

[28] Long F., & Aziz N. A. (2021). Travel Abroad for Face Gaining or Face Saving? A Comparison Between Chinese Gen Y Male and Female Tourists in a Context of Chinese Culture. Journal of International Consumer Marketing. doi: 10.1080/08961530.2021.1899882

[29] Wang Z., Guo D., Wang X., Zhang B., & Wang B. (2018). How does information publicity influence residents’ behaviour intentions around e-waste recycling?. Resources, conservation and recycling, 133, 1–9.

[30] Arnould E. J., & Thompson C. J. (2005). Consumer culture theory (CCT): Twenty years of research. Journal of consumer research, 31(4), 868–882.

[31] Han C. M., Wang X., & Nam H. (2020). The changing nature of consumer animosity and cosmopolitanism among young, individualistic consumers in emerging Asia: evidence from China. Asia Pacific Journal of Marketing and Logistics, 33(2), 647–666. doi: 10.1108/APJML-11-2019-0635

[32] Kozinets R. V. (2002). Can consumers escape the market? Emancipatory illuminations from burning man. Journal of Consumer research, 29(1), 20–38.

[33] Steenkamp J. B. E. (2019). Global versus local consumer culture: Theory, measurement, and future research directions. Journal of International Marketing, 27(1), 1–19.

[34] Hungara A., & Nobre H. (2021). A consumer culture theory perspective of the marketplace: An integrative review and agenda for research. International Journal of Consumer Studies. doi: 10.1111/ijcs.12670

[35] Dogan M., & Yaprak A. (2017). Self-construal and willingness to purchase foreign products: The mediating roles of consumer cosmopolitanism and ethnocentrism. In Creating marketing magic and innovative future marketing trends (pp. 1499–1511). Springer, Cham.

[36] Sharma P. (2015). Consumer ethnocentrism: Reconceptualization and cross-cultural validation. Journal of International Business Studies, 46(3), 381–389. doi: 10.1057/jibs.2014.42

[37] Sumner W.G. (1906), Folkways: The Sociological Importance of Usages, Manners, Customs, Mores, and Morals. New York: Ginn & Co.

[38] Nguyen N. T., & Pham T. N. (2021). Consumer attitudinal dispositions: A missing link between socio-cultural phenomenon and purchase intention of foreign products: An empirical research on young Vietnamese consumers. Cogent Business & Management, 8(1), 1884345. doi: 10.1080/23311975.2021.1884345

[39] Sun Y., Gonzalez-Jimenez H., & Wang S. (2021). Examining the relationships between e-WOM, consumer ethnocentrism and brand equity. Journal of Business Research, 130, 564–573.

[40] Shimp T. A., & Sharma S. (1987). Consumer ethnocentrism: Construction and validation of the CETSCALE. Journal of marketing research, 24(3), 280–289.

[41] Zeren D., Kara A., & Arango Gil A. (2020). Consumer ethnocentrism and willingness to buy foreign products in emerging markets: Evidence from Turkey and Colombia. Latin American Business Review, 21(2), 145–172.

[42] Blazquez-Resino J. J., Gutierrez-Broncano S., Jimenez-Estevez P., & Perez-Jimenez I. R. (2021). The Effect of Ethnocentrism on Product Evaluation and Purchase Intention: The Case of Extra Virgin Olive Oil (EVOO). Sustainability, 13(9), 4744. doi: 10.3390/su13094744

[43] Sousa A., Nobre H., & Farhangmehr M. (2018). The influence of consumer cosmopolitanism and ethnocentrism tendencies on the purchase and visit intentions towards a foreign country. International Journal of Digital Culture and Electronic Tourism, 2(3), 175–184.

[44] Qing P., Lobo A., & Chongguang L. (2012). The impact of lifestyle and ethnocentrism on consumers’ purchase intentions of fresh fruit in China. Journal of Consumer Marketing, 29(1), 43–51. doi: 10.1108/07363761211193037

[45] Thomas T., Singh N., & Ambady K. G. (2020). Effect of ethnocentrism and attitude towards foreign brands in purchase decision. Vision, 24(3), 320–329.

[46] Xin L., & Seo S. S. (2019). The role of consumer ethnocentrism, country image, and subjective knowledge in predicting intention to purchase imported functional foods. British Food Journal, 122(2), 448–464. doi: 10.1108/BFJ-05-2019-0326

[47] Chakraborty S., & Sadachar A. (2020). Predicting Indian consumers’ purchase intention from Western apparel brands. Journal of Fashion Marketing and Management: An International Journal, 25(3), 407–429. doi: 10.1108/JFMM-02-2020-0017

[48] Han C. M., & Guo C. (2018a). How consumer ethnocentrism (CET), ethnocentric marketing, and consumer individualism affect ethnocentric behavior in China. Journal of Global Marketing, 31(5), 324–338.

[49] Riefler P., Diamantopoulos A., & Siguaw J. A. (2012). Cosmopolitan consumers as a target group for segmentation. Journal of International Business Studies, 43(3), 285–305.

[50] Han C. M., & Won S. B. (2018b). Cross-country differences in consumer cosmopolitanism and ethnocentrism: A multilevel analysis with 21 countries. Journal of Consumer Behaviour, 17(1), e52–e66. doi: 10.1002/cb.1675

[51] Srivastava A., Gupta N., & Rana N. P. (2021). Influence of consumer cosmopolitanism on purchase intention of foreign vs local brands: a developing country perspective. International Journal of Emerging Markets, ahead-of-print (ahead-of-print). doi: 10.1108/IJOEM-01-2021-0057

[52] Gupta S., Nawaz N., Alfalah A. A., Naveed R. T., Muneer S., & Ahmad N. (2021). The Relationship of CSR Communication on Social Media with Consumer Purchase Intention and Brand Admiration. Journal of Theoretical and Applied Electronic Commerce Research, 16(5), 1217–1230. doi: 10.3390/jtaer16050068

[53] Singh K., & Misra M. (2021). Linking corporate social responsibility (CSR) and organizational performance: The moderating effect of corporate reputation. European Research on Management and Business Economics, 27(1), 100139. doi: 10.1016/j.iedeen.2020.100139

[54] Franco S., Caroli M. G., Cappa F., & Del Chiappa G. (2020). Are you good enough? CSR, quality management and corporate financial performance in the hospitality industry. International Journal of Hospitality Management, 88, 102395. doi: 10.1016/j.ijhm.2019.102395

[55] Duthler G., & Dhanesh G. S. (2018). The role of corporate social responsibility (CSR) and internal CSR communication in predicting employee engagement: Perspectives from the United Arab Emirates (UAE). Public relations review, 44(4), 453–462.

[56] Sharma V., Poulose J., Mohanta S., & Antony L. E. (2018). Influence of the dimensions of CSR activities on consumer purchase intention. Innovative Marketing, 14(1), 23–32.

[57] Sagheb M. Z., Ghasemi B., & Nourbakhsh S. K. (2020). Factors affecting purchase intention of foreign food products: An empirical study in the Iranian context. British Food Journal, 122(5), 1485–1504. doi: 10.1108/BFJ-05-2019-0318

[58] Jung H. J., Choi Y. J., & Oh K. W. (2020). Influencing factors of Chinese consumers’ purchase intention to sustainable apparel products: Exploring consumer “attitude–behavioral intention” gap. Sustainability, 12(5), 1770. doi: 10.3390/su12051770

[59] Churchill G. A. Jr (1979). A paradigm for developing better measures of marketing constructs. Journal of marketing research, 16(1), 64–73.

[60] Podsakoff P. M., MacKenzie S. B., & Podsakoff N. P. (2012). Sources of method bias in social science research and recommendations on how to control it. Annual review of psychology, 63, 539–569. doi: 10.1146/annurev-psych-120710-100452 21838546

[61] Maxwell-Smith M. A., White T. B., & Loyd D. L. (2020). Does perceived treatment of unfamiliar employees affect consumer brand attitudes? Social dominance ideologies reveal who cares the most and why. Journal of Business Research, 109, 461–471.

[62] Brislin R. W. (1970). Back-translation for cross-cultural research. Journal of cross-cultural psychology, 1(3), 185–216.

[63] Law W. W., & Xu S. (2017). Social change and teaching and learning citizenship education: An empirical study of three schools in Guangzhou, China. Citizenship Teaching & Learning, 12(1), 7–41.

[64] Reinartz W., Haenlein M., & Henseler J. (2009). An empirical comparison of the efficacy of covariance-based and variance-based SEM. International Journal of research in Marketing, 26(4), 332–344.

[65] Hair J. F., Black W. C., Babin B. J. & Anderson R. E. (2010). Multivariate data analysis (7th ed.). Upper Saddle River, New Jersey: Pearson Educational International.

[66] Hair J. F., Hult G. T. M., Ringle C. M., & Sarstedt M. (2021). A primer on partial least squares structural equation modeling (PLS-SEM). Sage publications.

[67] Dijkstra T. K., & Henseler J. (2015). Consistent partial least squares path modeling. MIS quarterly, 39(2), 297–316.

[68] Hair J. F., Risher J. J., Sarstedt M., & Ringle C. M. (2019). When to use and how to report the results of PLS-SEM. European business review, 31(1), 2–24. doi: 10.1108/EBR-11-2018-0203

[69] Kock N., & Lynn G. (2012). Lateral collinearity and misleading results in variance-based SEM: An illustration and recommendations. Journal of the Association for information Systems, 13(7). https://ssrn.com/abstract=2152644

[70] Cohen J. (1988). Statistical power analysis for the behavior science (2nd ed.). Hillsdale, NJ: Lawrance Eribaum association.

[71] Preacher K. J., & Hayes A. F. (2008). Asymptotic and resampling strategies for assessing and comparing indirect effects in multiple mediator models. Behavior research methods, 40(3), 879–891. doi: 10.3758/brm.40.3.879 18697684

[72] Wang Z. (2015). The Imbalance in Regional Economic Development in China and Its Reasons. In Private Sector Development and Urbanization in China (pp. 53–75). Palgrave Macmillan, New York.

