# Expression of Concern: Predicting young Chinese consumers’ intentions to purchase Western brands: Structural model analysis

**DOI:** 10.1371/journal.pone.0326797

**Published:** 2025-06-23

**Authors:** The PLOS One Editors

The *PLOS One* Editors issue this Expression of Concern to inform readers of concerns about aspects of the article’s peer review, including that there was a potential competing interest between the authors and one or more people involved in peer review. We regret that the issues were not identified prior to the article’s publication.
